# *In vivo *UVA irradiation of mouse is more efficient in promoting pulmonary melanoma metastasis than *in vitro*

**DOI:** 10.1186/1475-2867-11-16

**Published:** 2011-06-06

**Authors:** Riikka Pastila, Sirpa Heinävaara, Lasse Ylianttila, Dariusz Leszczynski

**Affiliations:** 1 Non-Ionizing Radiation Laboratory, STUK-Radiation and Nuclear Safety Authority, Laippatie 4, FIN-00880, Helsinki, Finland; 2 Health Risks and Radon Safety Laboratory; STUK-Radiation and Nuclear Safety Authority, Laippatie 4, FIN-00880, Helsinki, Finland; 3 Radiation Biology Laboratory; STUK-Radiation and Nuclear Safety Authority, Laippatie 4, FIN-00880, Helsinki, Finland

**Keywords:** B16, mouse melanoma, metastasis, UVA radiation, immunosuppression

## Abstract

**Background:**

We have previously shown *in vitro *that UVA increases the adhesiveness of mouse B16-F1 melanoma cells to endothelium.

We have also shown *in vivo *that UVA exposure of C57BL/6 mice, *i.v*. injected with B16-F1 cells, increases formation of pulmonary colonies of melanoma. The aim of the present animal study was to confirm the previously observed *in vivo *UVA effect and to determine whether *in vitro *UVA-exposure of melanoma cells, prior the *i.v*. injection, will have an enhancing effect on the pulmonary colonization capacity of melanoma cells. As a second aim, UVA-derived immunosuppression was determined.

**Methods:**

Mice were *i.v*. injected with B16-F1 cells into the tail vein and then immediately exposed to UVA. Alternatively, to study the effect of UVA-induced adhesiveness on the colonization capacity of B16-F1 melanoma, cells were *in vitro *exposed prior to *i.v*. injection. Fourteen days after injection, lungs were collected and the number of pulmonary nodules was determined under dissecting microscope. The UVA-derived immunosuppression was measured by standard contact hypersensitivity assay.

**Results and Discussion:**

Obtained results have confirmed that mice, *i.v*. injected with B16-F1 cells and thereafter exposed to UVA, developed 4-times more of melanoma colonies in lungs as compared with the UVA non-exposed group (p < 0.01). The *in vitro *exposure of melanoma cells prior to their injection into mice, led only to induction of 1.5-times more of pulmonary tumor nodules, being however a statistically non-significant change. The obtained results postulate that the UVA-induced changes in the adhesive properties of melanoma cells do not alone account for the 4-fold increase in the pulmonary tumor formation. Instead, it suggests that some systemic effect in a mouse might be responsible for the increased metastasis formation. Indeed, UVA was found to induce moderate systemic immunosuppression, which effect might contribute to the UVA-induced melanoma metastasis in mice lungs.

## Background

Ultraviolet-B radiation (UVB, 280-320 nm) is known for its harmful DNA-damaging potential and carcinogenic effects [1]. Ultraviolet-A radiation (UVA, 320-400 nm) has also been shown to damage DNA [2-4], promote carcinogenesis, and participate in the pathogenesis of squamous cell carcinoma [4-6]. Some studies have implicated UVA in the development of cutaneous melanoma [7,8], whereas others found it as ineffective [9]. In addition to UV's carcinogenic effects, both UVB and UVA radiation have been shown to induce local and systemic immunosuppression [10-13]. UV-derived immunosuppression has been identified as a risk factor for skin cancer induction because it allows DNA-damaged cells to survive and proliferate by escaping from the surveillance of the immune system [14].

The possibility that UV radiation may affect melanoma metastasis has not been addressed widely, although some UV-derived biochemical responses, like above-mentioned systemic immunosuppression or altered adhesion molecule expression [15] can possibly enhance tumor metastasis. We have previously shown in a small scale pilot study that UVA causes an increase in the pulmonary tumor formation in mice after intravenous inoculation of mouse melanoma B16-F1 cells [16]. Pulmonary colonization of the B16 cells after *i.v*. injection is a metastasis-like event resembling the hematogenous metastasis of melanoma and gives an indication of their metastatic potential, as shown by Fidler [17,18]. We have also demonstrated that UVA exposure causes the increase in the adhesiveness melanoma cells to endothelium *in vitro*, what is accompanied by changes in the expression of the cell surface adhesion molecules [19]. Thus, we proposed that the UVA-derived changes in the expression of adhesion molecules could be, at least in part, responsible for the *in vivo *observed increase of pulmonary tumor colonies in mice.

The aim of the present animal study was to confirm the previously observed *in vivo *UVA-induced increase in the melanoma tumor formation in mice lungs, and to determine whether *in vitro *UVA-exposure of melanoma cells will have similar enhancing effect on the pulmonary colonization capacity of melanoma. The obtained results postulate that *in vivo *irradiation of mice has more significant effect on the pulmonary melanoma metastases formation than *in vitro *irradiation of cells, suggesting that some systemic effect in a mouse, rather than a direct effect of the UVA on the melanoma cells, might be responsible for the increased metastasis formation. One possible systemic effect could be the UV-induced immunosuppression.

## Methods

### UVA radiation source and dosimetry

A facial tanner lamp, Philips HB 171/A (Philips, Germany) with four Philips Cleo 15 W lamps, was used as a radiation source. UVB radiation was filtered out with a 5 mm thick glass filter. The spectral irradiances were measured with a temperature stabilized Bentham DM150 (Bentham Instruments Ltd., Berkshire, England) double-monochromator spectroradiometer at 0.5 nm intervals from 250 nm to 400 nm as described previously [16,19].

**Figure 1:**
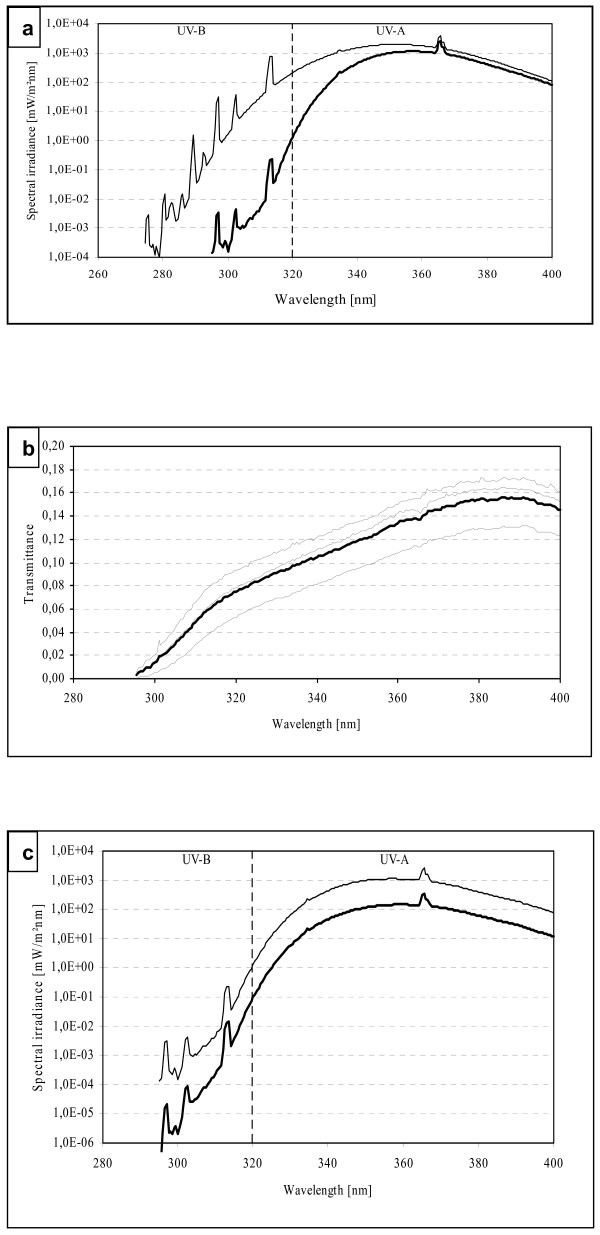
**The spectral irradiance and spectral transmittance of UVA radiation**. **(a) **The spectral irradiance of a Philips HB 171/A facial tanner is indicated with a thin line. The irradiance with a glass plate filter, cutting off the UVB portion, is indicated with thick line. **(b) **The transmittance of three mice skins, measured *ex vivo*, is shown with 3 thin lines, respectively. The average transmittance is indicated by a thick line. **(c) **The actual spectral irradiance transmitted through mice skin (thick line) was determined by multiplying the measured spectral irradiance of Philips facial solarium (thin line) with the average transmittance (thick line in Figure 1b).

The irradiances in both *in vitro *and *in vivo *irradiation set-ups were measured at the same distance from the lamp as in the actual UV-exposures. The attenuation caused by the Petri dish lid and the culture medium was taken into account in the *in vitro *experiments by measuring the irradiance through dish cover and the culture medium (data not shown). The irradiation spectra were almost identical in both set-ups consisting of 99.99% UVA and 0.01% UVB. The spectrum used in the *in vivo *irradiations is shown in Figure 1a, in which UVA spectrum irradiating mice is indicated with a thick line after filtration through a 5 mm glass plate.

### The transmittance of mouse skin

The transmittance of C57BL/6 mouse skin was measured *ex vivo *using the Bentham DM 150 spectroradiometer (Bentham Instruments Ltd., Berkshire, England). A Philips HP 3136 sun lamp (Philips, The Netherlands) was used as a irradiation source. Although its irradiance is higher as compared to Philips HB 171/A facial solarium used in the *in vivo *mice UVA irradiations, the transmission through mice skin remains the same.

Hair on the mice abdomens was shaven off, after which animals were euthanized and skin was removed. The location and the size of shaved and removed skin were identical to that used during *in vivo *UVA exposures.

The lamp spectrum was determined first, after which spectral measurements were performed on three abdominal skins. The spectral transmittance was calculated by dividing the spectrum obtained through mouse skin by the spectrum of the Philips HP 3136 sun lamp (Figure 1b). The actual spectral irradiance transmitted through the mouse skin during the UVA *in vivo *exposures was determined by multiplying the average spectrum of transmission (Figure 1b, thick line) with the spectrum of the used Philips HB 171/A face solaria (Figure 1c, thin line). The spectrum indicated with the thick line in Figure 1c depicts the spectral irradiance transmitted through mouse skin during the *in vivo *UVA irradiation experiments.

### Cells

C57BL/6 mice-derived melanoma cell lines B16-F1 and B16-F10 were purchased from National Cancer Institute, Frederick Cancer Research & Development Center (Frederick, MD, USA). Melanoma cell lines were grown in RPMI-1640 and the cell culture media were supplemented with 10% heat-inactivated FBS, penicillin (100 IU/ml), streptomycin (100 μg/ml), and L-glutamine (4 mM). All cell culture supplies were purchased from Gibco BRL, Paisley, UK. Prior to injection melanoma cells were harvested with the EDTA cell stripper solution (140 mM NaCl, 2.7 mM KCl, 8 mM Na_2_HPO_4_, 0.5 mM EDTA) to preserve surface adhesion molecules. Thereafter, the melanoma cells were washed once and 50.000 melanoma cells were suspended in 0.2 ml of 0.9% NaCl for injection.

### Animals

Female C57BL/6 (C57BL/6JOlaHSd) mice, Specific Pathogen Free (SPF) status according to Felasa Health Monitoring Guidelines, were purchased from Harlan Laboratories, The Netherlands, and housed in Viikki Laboratory Animal Center, University of Helsinki, Finland. The ethical evaluations of experiments were reviewed and approved by the Institutional Animal Use and Care Committee of the University of Helsinki and The State Provincial Veterinarian Office of Southern Finland. The care, welfare and use of the animals were in accordance with national, institutional and European guidelines. Mice were at 8-10 weeks of age at the beginning of the experiments. There were no pregnant or lactating animals among the study subjects. Mice were housed and arranged for the experiments to the groups of five animals, with free access to water and food.

Prior to the experimental procedures mice were anesthetized using a combination of a neuroleptanalgesic drug Hypnorm (fentanyl citrate at 0.315 mg/ml and fluanisone at 10 mg/ml; Janssen Pharmaceutical, Tilburg, The Netherlands), and a sedative Dormicum (midazolam 1 mg/ml, Roche, Basel, Switzerland). Mice were anesthetized with intraperitoneal (*i.p*.) injection of a mixture of Hypnorm-Dormicum diluted 1:1 vol/vol, and administered 0.05-0.075 ml per animal.

### Melanoma cell injection and *in vivo *UVA irradiation of mice

**Figure 2:**
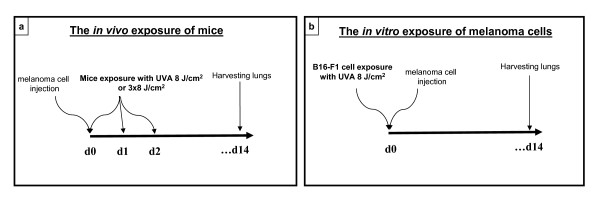
**Time-line of the animal study**. **(a) **Mice were injected with B16-F1 cells into the tail vein and immediately after injection exposed *in vivo *to one, or three consecutive, dose of UVA. **(b) **B16-F1 cells were *in vitro *exposed to the single dose of 8 J/cm^2 ^UVA before injection into the tail vein. In the both experimental set-ups, fourteen days after melanoma cell injection mice lungs were removed, fixed, and melanoma nodules were counted on the lung surface.

All mice groups consisted of ten mice. The non-exposed control mice were *i.v*. injected into the tail vein by the suspension of 50.000 of B16-F1 (low-metastatic) or B16-F10 (high-metastatic) cells in 0.2 ml of saline (Figure 2a), as described previously [16]. Immediately after cell injection mice were sham-treated. In the UV-treatment group, hair on the abdomens was shaven off to allow UVA irradiation of skin, and mice were *i.v *injected into the tail vein by the suspension of 50.000 of B16-F1 cells in 0.2 ml of saline (Figure 2a). Immediately following the B16-F1 cell injection, the abdominal side of mice was exposed to a single UVA dose at 8 J/cm^2^. Some of the animals were exposed to two more UVA doses (8 J/cm^2^) at 24 and 48 hours after the melanoma cell injection on two consecutive post-injection days. Mice in all treatment groups were terminated 14 days after melanoma injection, lungs were removed and fixed in Bouin's solution for 48 hours, after which the tumor colonization in lungs was evaluated.

### *In vitro *UVA irradiation of cells prior to their injection

The B16-F1 cells were exposed in the RPMI 1640 medium to the single UVA dose of 8 J/cm^2^, as described previously [19]. The irradiation was performed at room temperature in a dark room on a black support to avoid effects of reflected radiation. The non-irradiated control B16-F1 and B16-F10 cells were sham-treated by keeping at room temperature in a dark room for the irradiation time. After UVA exposure or sham treatment, the cells were washed carefully twice with PBS, after which the suspension of 50.000 of B16-F1 or B16-F10 cells in 0.2 ml of saline was *i.v*. injected into the tail vein of C57BL/6 mice (Figure 2b). Mice were terminated 14 days after injection and the lungs were removed and fixed in Bouin's solution for 48 hours, after which the tumor colonization in lungs was evaluated.

### The evaluation of the pulmonary metastases

**Figure 3:**
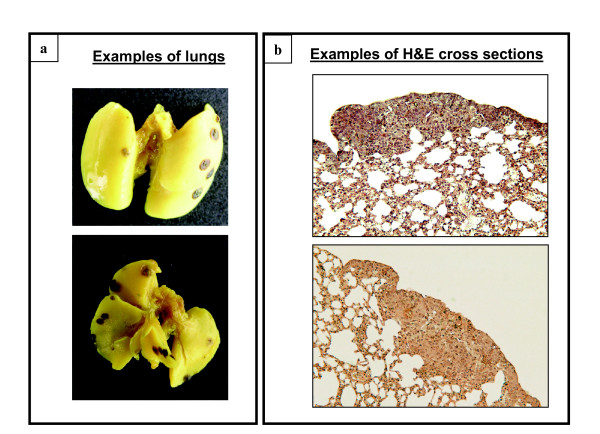
**Representative examples of the lungs and of the lung cross sections after UVA radiation and B16-F1 cell injection**. **(a) **Metastatic nodules, which were clearly visible on the lung surface, were counted under dissecting microscope (magnification of the objective 6×). **(b) **Lung cross sections (5 μm) were stained with H&E to determine the lung tissue morphology and the appearance of tumors (magnification of the objective 10×).

The quantitative evaluation of the pulmonary tumor nodules was performed under dissecting microscope (Wild Heerbrugg AG, Switzerland) by counting the clearly visible and easily detectable tumors on the lung surface (Figure 3a), as described previously [16]. Thereafter lungs were embedded in paraffin, cut using a rotary microtome (Microm International GmbH, Walldorf, Germany) in 5 μm-sections, stained with standard hematoxyline-eosine method and examined for the presence of the invisible micrometastases in tissue parenchyma under dissecting microscope (Figure 3b).

Statistical analysis of pulmonary tumor formation was performed using non-parametric the Wilcoxon-Mann-Whitney (rank sum) test between *in vivo *UVA-exposed mice group and non-exposed mice group as well as between the mice group treated either *in vitro *UVA-exposed melanoma cells or the sham-treated cells. P values were adjusted for multiple comparisons at significance level of 0.05.

### Contact Hypersensitivity (CHS) assay

**Figure 4:**
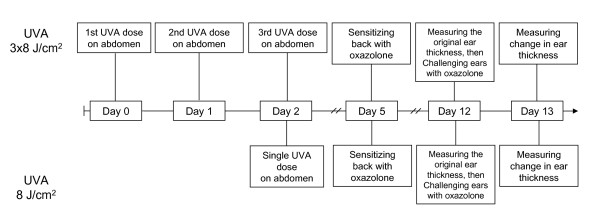
**Time-line of the CHS assay showing the order of procedures**. Mice were irradiated with a single dose of UVA or consecutive doses of UVA in three days. Three days later from the latest UVA dose, mice were sensitized to the shaved back with oxazolone. Mice were challenged with oxazolone to both ear pinna 7 days after sensitization. The thickness of the ears, i.e. ear swelling, was measured 24 hours after challenge.

In a pilot CHS-assay, each group consisted of ten mice. In the repetition of the CHS assay, each group consisted of 27 mice to obtain statistically more powerful analysis. Experimental design showing time line and order of procedures are shown in Figure 4. Mice were irradiated on shaved abdomen with UVA at 8 J/cm^2 ^(group A). Group A had its own irritant control mice (group C), which was treated similarly as group A, but not sensitized. The positive control group for CHS formation (group B) was not UVA-exposed and it had its own non-sensitized control group D. During UVA-exposure, mice heads were covered with a loosen "cap", made from black plastic electrical tape, to avoid the UV-mediated alterations in the CHS response, when challenging ears 10 days later. This protective cap did not interact with the ear skin, thus causing any potential irritation on the skin, or did not prevent normal respiration of the animals.

Three days after the UVA/sham treatment, 2% oxazolone (4-ethoxymethylene-2-phenyl-2-oxazolin-5-one, dissolved and diluted in acetone) (Sigma Chemical Co., St. Louis, MO, USA), was applied as a sensitizer (100 μl, corresponding 2 mg of oxazolone) to the shaved back of mice that received UVA radiation (group A) as well as of the CHS positive control mice (group B) under anesthesia. The sensitization procedure was not performed to the irritant controls groups C and D.

The challenging dose of the sensitizer (10 μl, corresponding 200 μg of oxazolone) was applied to both ear pinna 7 days after sensitization for all groups. The thickness of the ears was measured before application and 24 h later with Pocket Thickness Gage No. 7309 (Mitutoyo Corp., Japan). Thickness of the ears was measured by two persons, both measuring their own mice, and the same number of mice from all the treatment groups. The change of the ear thickness was calculated from both ears by subtracting the original thickness from the challenged ear thickness and taking the average from these two values. Finally, the UVA induced suppression *s *was calculated according to the formula (1) [20]. A, B, C, and D are the average swelling figures for the respective animal groups 24 hours after challenge as follows:(1)

Statistical significance of UVA immunosuppression was analyzed using the analysis of covariance. The average change in the ear thickness was first explained by the group (A, B, C, D) and the average original thickness as covariate. The average change was analyzed further by including the measurer in the model. A priori, the average difference in the effect of B-D was assumed to be larger than that of A-C based on results obtained from a pilot CHS assay. The average difference between these effect differences was therefore assessed at one-sided significance level of 0.05.

## Results

### The majority of UVA was absorbed in the mouse skin

The transmittance of the UVA wavelengths through mice skin (n = 3) varied from 5% to 15% as shown in Figure 1b, indicating that the majority of the UVA radiation, approximately 90%, was absorbed in the mouse skin. The actual spectral irradiance transmitted through mice skin during the *in vivo *irradiation is shown with the thick line in Figure 1c.

### *In vivo *UVA irradiation of mouse increased remarkably the pulmonary metastasis formation

*In vivo *UVA irradiation of mice that were injected with low-metastatic B16-F1 melanoma prior to the exposure caused a remarkable increase in the number of pulmonary metastases. Animals exposed to a single dose of UVA (Table 1, group 1c) developed 4-times more metastases as compared to the non-irradiated mice injected with the B16-F1 cells (Table 1, group 1a). This difference was statistically significant (p < 0.01).

**Table 1:** The quantitative evaluation of the pulmonary melanoma nodules on the lung surface.

Treatment^1^	Tumors		Treatment^2^	Tumors	
*In vivo*	Number per ten mice	Median	*In vitro*	Number per ten mice	Median
**1a: **B16-F1	27	2	**2a: **B16-F1	22	2

**1b: **B16-F10	70	4.5	**2b: **B16-F10	82	5

**1c: **B16-F1 + UVA	117*	7.5	**2c: **B16-F1 + UVA	34	2

**1d: **B16-F1 + 3 × UVA	72	4	-	-	-

^1 ^C57BL/6 mice (n = 10) were *in vivo *irradiated after *i.v*. melanoma cell injection into the tail vein. *p < 0.01

^2 ^B16-F1 melanoma cells were *in vitro *irradiated before their *i.v*. injection into the tail vein of C57BL/6 mice (n = 10).

The effect of three repetitive UVA exposures had weaker effect on the metastatic potential of B16-F1 cells than the single UVA exposure. Although there was a 2.5-fold increase in the metastases formation (Table 1, group 1d) as compared to the non-exposed controls (Table 1, group 1a), it was statistically non-significant. However, the number of metastases remained high on the lung surface after three doses of UVA and reached the same level than the positive controls animals, injected with high-metastatic B16-F10 cells (Group 1b, Table 1).

### *In vitro *irradiation of B16-F1 cells had a minor effect on the metastatic potential

*In vitro *UVA irradiation of B16-F1 cells prior to the cell injection caused a small, only 1.5-fold increase in the number of pulmonary metastases (Table 1, group 2c) as compared to animals that were injected with the non-irradiated B16-F1 cells (Table 1, group 2a). Although this change was ca. 50% increase in tumor formation, it was not statistically significant.

### UVA radiation caused a moderate immunosuppression determined by CHS

The pilot CHS assay (n = 10 mice/group) showed a non-significant 13.8% increase in immunosuppression in UVA irradiated mice (data not shown). Irradiation of mice with three consecutive UVA doses neither induced decline nor increase in the CHS effect (data not shown).

**Figure 5:**
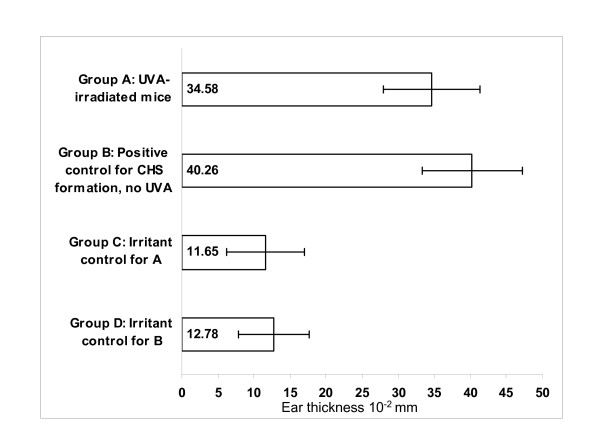
**UVA-derived moderate immunosuppression after single dose of UVA**. Changes in the ear thickness as a measure of immunosuppression are shown in mice irradiated with a single dose of UVA (group A) as compared with the non-irradiated, positive control group for CHS formation (group B). Groups C and D are the irritant (non-sensitized) controls for UVA treated mice (A), and for non-irradiated mice (B), respectively. Error bars depict standard deviations.

The repetition of CHS assay (n = 27 mice/group) showed an increase in immunosuppression by 16.6% (p = 0.0265, one-sided) after the single dose of UVA. In the positive control group for CHS formation (group B, Figure 5), the mean ear swelling was 27.5 × 10^-2^mm after subtracting the background (group D). In the UVA-irradiated mouse group (group A), mean ear swelling declined to 22.9 × 10^-2^mm, after background subtraction (group C). The average ear thickness change of each mouse was calculated using measures for each ear thickness pre- and post-challenge. Two persons measured thickness of the ears and the both measurers found the same trend in the ear swelling figures, but differences existed between the measurers.

## Discussion

In this study we confirmed our previous finding that UVA increases the pulmonary colonization capacity of mouse melanoma [16] and demonstrated that a single UVA exposure of mice with *i.v*. injected B16-F1 melanoma cells caused statistically significant, 4-fold increase in the metastasis in terms of pulmonary colonization. Exposing the B16-F1 *i.v*. injected animals to UVA radiation on three consecutive days, the number of pulmonary metastases remained high, but the result was statistically non-significant as compared to the non-irradiated controls. Nevertheless, the tumor formation capacity was comparable with the number of pulmonary metastases formed by highly-metastatic B16-F10 cells.

The transmittance of the mouse skin was measured to determine how much the UVA radiation attenuates when interacting with the skin tissue, and whether UVA reaches the dermis and thus, presumably the circulating melanoma cells in the capillary network. In the human skin UVA radiation is able to penetrate to the upper dermis. Mouse epidermis is approximately 10 μm and for dermis 250 μm [21], being remarkably thinner as compared to human skin (1-4 mm). In this study, approximately 90% of the used UVA wavelengths were absorbed in the skin and ~10% was transmitted through it. Thus, UVA had a possibility to affect the epidermal cells, such as keratinocytes and mast cells, which are known to be rich source of immunologically active compounds [11,12], but also affect the cells in the dermis.

Previously we have hypothesized that the UVA-induced changes in the melanoma adhesiveness, and in the adhesion molecule expression *in vitro *[19], might be factors that could be responsible for the increased metastatic potential *in vivo*. To validate this hypothesis in the current study, the low-metastatic B16-F1 cells were *in vitro *exposed with a single UVA dose at 8 J/cm^2 ^prior to the cell injection. This treatment increased the metastatic capacity of melanoma cells only by 1.5-fold, being a statistically non-significant change. The apoptosis or necrosis of the cells after *in vitro *UVA radiation cannot explain the overall lower metastatic potential of the B16-F1 melanoma, since either the used UVA dose [19], or RPMI media during irradiations (unpublished finding), were not found to cause any adverse cellular effects or cell death. Furthermore, the *in vitro *or *in vivo *irradiation modality did not seem to cause any differences in the location or morphology of the metastases: histological evaluation of haematoxylin-eosin stained tissue revealed that the vast majority of the tumors were visible on the lung surface and metastases were rarely seen deep inside the lung parenchyma regardless the used UV delivery method. This is also in accordance with our pilot histology analysis [16], which showed that the majority of the metastases were seen on the lung surface.

This result obtained here suggests that other mechanism(s), than the UVA-induced endothelial adhesiveness of melanoma cells, might play a role in the UVA-induced increase of the pulmonary metastasis formation. One such mechanism could be the UV-induced systemic immunosuppression that might help in the progression of metastasis by impairing the melanoma cell rejection in the UV-exposed mice, in a similar way how the drug-mediated immunosuppression has been shown to enhance the skin tumor invasion in the patients undergoing organ transplantation [22,23]. UV-derived immunosuppression was first associated with UVB radiation, and is utilized widely in the UVB-phototherapy treatments for a variety of different dermatoses [24,25].

In our current study the UVA-induced systemic immunosuppression was measured by the contact hypersensitivity assay that has been shown to be an informative and useful approach to determine the UV-induced immune responses in mice [26]. Both our CHS assays showed the same trend; the single UVA dose at 8 J/cm^2 ^caused a moderate increase in systemic immunosuppression as compared to the non-exposed control animals. This finding was in accordance with Byrne et al., who has also shown that UVA irradiation suppressed the systemic contact hypersensitivity in C57BL/6 mice [10]. They have also shown that in contrast to primary UVA-induced immunosuppression, further UVA irradiation of C57BL/6 mice enhanced the secondary immune responses. Our current result agrees with Byrne's finding since the immunosuppression of systemic contact hypersensitivity was not present anymore when the three UVA doses were applied in our pilot CHS study. In our model, the consecutive UVA exposures might have had an anti-immunosuppressive effect offering an explanation for the observation that three exposures delivered on the three consecutive days, had weaker pro-metastatic effect than the single dose in terms of pulmonary metastases formation (data-not shown).

The immunosuppression data presented in this study do not, however, show whether UVA is directly involved in the decline of the cellular immunity against tumor cells, and it needs to be confirmed in the future studies. This data only demonstrates that UVA enables the melanoma metastasis in mice, possibly in the similar manner that UV-mediated immunosuppression enables the transplanted tumors to grow in the UV-treated mice, in which tumor growth was only apparent, if the recipient mice were first UV exposed before cell injection, whereas the non-exposed recipients were able to reject the tumor cells [27,28].

## Conclusion

UVA irradiation is capable to enhance the pulmonary metastasis in a mouse model, but our results suggest that some UVA-induced systemic effect(s) in mice plays more prominent role in the enhanced melanoma metastasis than the direct UVA-induced increase in adhesiveness of melanoma cells. UVA was found to induce moderate systemic immunosuppression, which effect might contribute to the UVA-induced melanoma metastasis. The used UVA dose of 8 J/cm^2 ^roughly corresponds to the UVA dose received approximately within 1 hour on a sunny summer day in Finland, thus being physiologically relevant also for humans. These results presented in this study suggest that, if occurring also in humans, exposure to UVA radiation during extensive sunbathing or solaria tanning periods might have the potential to cause increase in the haematogenous melanoma metastasis in patients with metastatic disease.

## List of abbreviations

UVA: Ultraviolet-A; CHS: contact hypersensitivity.

## Competing interests

The authors declare that they have no competing interests.

## Authors' contributions

RP designed the experimental set-up and carried out the experiments. SH made the statistical analysis of the data. LY designed the mouse irradiation chamber and performed UV dosimetry and transmittance measurements. DL participated in the study design and helped to draft the manuscript. All authors read and approved the final manuscript.
